# Rapid Detection of Amitriptyline in Dried Blood and Dried Saliva Samples with Surface-Enhanced Raman Spectroscopy

**DOI:** 10.3390/s22218257

**Published:** 2022-10-28

**Authors:** Ramin Boroujerdi, Richard Paul, Amor Abdelkader

**Affiliations:** Faculty of Science and Technology, Bournemouth University, Talbot Campus, Fern Barrow, Poole BH12 5BB, UK

**Keywords:** amitriptyline, dried blood spot (DBS), dried saliva spot (DSS), surface-enhanced Raman spectroscopy (SERS), toxicology

## Abstract

There is growing demand for rapid, nondestructive detection of trace-level bioactive molecules including medicines, toxins, biomolecules, and single cells, in a variety of disciplines. In recent years, surface-enhanced Raman scattering has been increasingly applied for such purposes, and this area of research is rapidly growing. Of particular interest is the detection of such compounds in dried saliva spots (DSS) and dried blood spots (DBS), often in medical scenarios, such as therapeutic drug monitoring (TDM) and disease diagnosis. Such samples are usually analyzed using hyphenated chromatography techniques, which are costly and time consuming. Here we present for the first time a surface-enhanced Raman spectroscopy protocol for the detection of the common antidepressant amitriptyline (AMT) on DBS and DSS using a test substrate modified with silver nanoparticles. The validated protocol is rapid and non-destructive, with a detection limit of 95 ppb, and linear range between 100 ppb and 1.75 ppm on the SERS substrate, which covers the therapeutic window of AMT in biological fluids.

## 1. Introduction

Amitriptyline (AMT) is a common and widely used tricyclic antidepressant (TCA) for patients with major depressive disorder [1,2]. Similar to other TCAs, AMT displays large individual differences in clearance, and the therapeutic window of AMT is relatively small (150 ppb–300 ppb) [3,4,5]; thus, therapeutic drug monitoring (TDM) is necessary. AMT is proven to induce long-term impact on central nervous systems by affecting the brain serotonin (5-HT), 5-hydroxyindoleacetic acid, norepinephrine, and acetylcholine levels, and it affects the locomotor activity (schooling behavior) [6,7]. Among all TCAs, only amitriptyline is linked to brain death by depressing brain stem functions [8], in addition to causing sudden death by cardiac arrest [9,10]. Due to its hazardous nature, AMT detection is important in medical investigation, forensic pathology, and toxicology [2,11,12]. Many suicidal death cases were found to be related to the toxic effects of AMT [13]. In addition to suicide, AMT is abused for its euphorigenic effects [11], which can lead to death. AMT has also shown synergistic effects when administrated orally with other medicines such as paracetamol [14], which increases the importance of its rapid and accurate measurement. In addition to the pharmacological and medical importance of measuring AMT, a reliable detection technique to detect AMT in water samples is required for environmental toxicology investigations. Due to the high worldwide level of consumption, AMT has entered the aquatic environment through human excretion, expired drug disposal, and medical wastewater [6,15,16]. However, AMT is not biodegraded in wastewater treatment plants; it remails biologically active and persistent in aquatic environments and is considered a threat to wildlife [17,18,19].

The collection of whole blood samples on paper is known as dried blood spot (DBS), which dates back to the 1960s [20] and is still in common use [21,22,23]. DBS offers several advantages over conventional whole blood, plasma, or serum sample collection such as requiring a less invasive sampling method (small needle prick, compared to venous cannula), easier and simpler storage and transfer (no need for freezing samples), and potential to be collected by patients themselves (minimal training); furthermore, it reduces the infection risk caused by various pathogens, and only requires a very small blood volume compared to conventional blood sample collection [24,25,26,27,28]. 

While most drugs are highly bound to blood proteins, it is only their free fraction which is pharmacologically active. In the case of AMT, its pK_a_ and LogP values are 9.40 and 4.94, respectively [3], which means that, depending on the blood pH, only about 1% of AMT that enters blood will be nonionized at any given time and can pass through the cell membranes. While the LogP value indicates its fat solubility, AMT can also dissolve in aqueous sample at low concentrations, and, when digested, it can lose a methyl group through the process of metabolism, turning into its metabolite, nortriptyline (the half-life of AMT is about 36 h) [1,7,29,30]. Saliva contains the free fraction of drugs that is released by salivary tissues and, therefore, provides a better indication of the therapeutically active fraction of drug and its state of intoxication [31,32,33]. Dried saliva spot (DSS) which has seen growing interest in the past decade is another method of collecting biological samples on paper. While DSS still holds some of the advantages of DBS, it also offers noninvasive sampling compared to blood sampling and is easier to collect and handle [33,34,35]. 

DBS and DSS have proven to be useful samples in which to measure various drugs and medicines including AMT. However, despite the significant advances in bioanalysis exploiting DBS and DSS, there are still some challenges that limit their application. Analytical techniques commonly employed for AMT analysis such as gas chromatography/mass spectrometry (GC/MS) or liquid chromatography/mass spectrometry (LC/MS), in addition to being costly and destructive (samples cannot be recovered), require expertise to operate, while sample pretreatment procedures are time-consuming [4,12,36]. 

Immunoassay, another commonly used technique in toxicological investigations [37,38,39], has also been used for TDM to speed up the analysis process in comparison to chromatography techniques while making onsite testing possible; however, the technique can be prone to false negatives, it may require some sample pretreatment, and response times can be slow [39,40,41,42]. A potential replacement method to tackle the issues of previous techniques in analyzing DSS and DBS could be surface-enhanced Raman spectroscopy (SERS).

Raman spectroscopy is a useful technique for the screening of drugs of abuse in biological matrices [43,44]. In Raman analysis, each chemical bond in the analyte requires a specific energy to vibrate; thus, a unique fingerprint pattern can be built from the different chemical bonds and compounds found in the sample. Detailed information obtained from Raman spectroscopy can lead to the detection of various specific constituents, such as toxins and drugs, within complex matrices [45,46,47]. However, conventional Raman spectroscopy may suffer from low sensitivity which prevents the detection of molecules with very low Raman efficiency, samples at very low concentrations, and small volumes or quantities of samples. Such drawbacks make accurate detection of target analytes from within a complex matrix highly challenging when using conventional Raman spectroscopy. Surface-enhanced Raman spectroscopy (SERS) has been developed and improved over the past decade to enhance the Raman signal up to 1015 times and has been proven to tackle the low sensitivity of conventional Raman spectroscopy [48]. Raman enhancement is achieved when the molecule of interest is in close proximity to a metallic surface with plasmonic properties or close to a metal nanoparticle [49,50]. The common nanoparticles which have been used in SERS analysis are gold and silver, and it has been demonstrated that the size and shape of these nanoparticles can also affect the outcome spectra (sensitivity) of SERS [49,51,52].

SERS paper-based substrates have been developed using various methods, such as the addition of metal nanoparticles to the paper via thermal inkjet printer, dipping, drop casting, or dripping on filter paper, damp paper, or common dry office paper [51,53,54,55]. Papers offer a 3D, porous, and heterogeneous morphology, and the way that filter paper adsorbs nanoparticles makes it a perfect candidate for SERS studies [55]. Papers, in addition to affordability, offer flexibility, efficient uptake, and absorption of liquid samples on the surface [51,56,57]. 

While the analysis of pure AMT crystals [58] and real-time AMT detection in liquid blood plasma [59] with Raman spectroscopy has been reported previously, no one to date has utilized this technique to investigate the detection of such drugs in dried biological samples. Here, we present the enhancement of dried blood and saliva spot tests with silver nanoparticles (AgNPs) for the first time, making the detection of AMT in dried biological fluids with Raman spectroscopy possible. Utilizing the SERS technique not only makes accurate and precise onsite testing possible (portable Raman spectroscope), but it also eliminates the pretreatment steps and can provide results in just a few seconds.

## 2. Experimental

### 2.1. Materials and Instrumentation

All chemicals were purchased from Fisher Scientific, UK, and were of analytical reagent grade. All aqueous solutions were prepared using double-distilled water. Human saliva and horse blood (TSC Biosciences, Botolph Claydon, UK; REF: HB041) were used as authentic biological samples for this study. In order to collect saliva, participants allowed saliva to pool in the mouth, and then deposited it in a centrifuge tube. Biological samples were kept in the fridge at 4 °C for the duration of this experiment. 

Fourier-transform infrared (FTIR) spectra were recorded on Platinum ATR—Alpha II FTIR spectrophotometer (Bruker, Billerica, MA, USA). Attenuated total reflection (ATR) sampling technique was used to study samples. After collecting the background spectra, a single drop of liquid sample or a 2 mm^2^ piece of solid sample was placed on the crystal with a high refractive index (at a set angle) of the ATR system, and the results of 24 scans for each sample were collected in a range between 600 cm^−1^ and 4000 cm^−1^, reported in the form of a single spectrum. The FTIR samples were collected from different sections of the sample, to ensure the reproducibility of the responses. Baselines were corrected with Origin software to make the comparison between peaks more reliable.

Scanning electron microscopy (SEM) and energy-dispersive X-ray analysis (EDAX) measurements were carried out using a JEOL JSM-6010 tungsten cathode scanning electron microscope. To prevent charge build-up while analyzing samples with a focused beam of high-energy electrons, dry solid samples were first fixed on a conductive carbon tape, and all samples were studied in a low-vacuum mode. Beam voltage for collecting SEM images was set to 10 kV, whereas, for EDAX, it was increased to 15 kV; the lower voltages were used while obtaining images to increase topographic contrast and minimize specimen charging and possible beam damage. 

An XploRA™ PLUS Raman spectrometer was used to collect Raman spectra and mapping. Solid samples were placed and fixed on a silicon surface before an image was obtained from the surface of the sample with a 50× objective. A 532 nm laser was chosen for excitation, and stronger lasers were avoided to prevent any possible damage to the biological samples. Hole size, slit size, gratings, and filters were set to result in the maximum signal-to-noise ratio for all tested samples while reducing the chances of reaching saturation levels early through slight changes in the concentration. A hole and slit size of 100 μm, 1200 T grating, and 50% filter were found to provide the best results on our samples for the 532 nm excitation laser (50× objective), when the Raman measurements were performed at 10 s acquisition time with 10 accumulations to analyze samples (at various spectrum ranges). Raman mapping was developed from analyzing 125 points in three dimensions on the surface of the AgNP-decorated filter paper sample to investigate the homogeneity of the substrate and reproducibility of responses.

### 2.2. AgNP Synthesis and Casting Procedure

Silver nanoparticles were prepared using the Lee and Meisel chemical reduction method [60] with modification. Briefly, 6 mL of 1 N AgNO_3_ was added to 250 mL of double-distilled water and heated to 70 °C while stirring vigorously. Then, 10 mL of 0.08 mM trisodium citrate solution was added to the mixture drop by drop over 15 min. Next, 30 min after the addition of trisodium citrate, the temperature was increased to boiling point, and the color of the solution started to slowly change from colorless to light yellow; eventually, after 30 min, it formed a cloudy solution. Heating and stirring were ceased, and the mixture was left to cool down to room temperature, while covered from the light. The synthesis reaction is illustrated in Equation (1).

4Ag^+^ + C_6_H_5_O_7_Na_3_ + 2H_2_O → 4Ag^0^ + C_6_H_5_O_7_H_3_ + 3Na^+^ + H^+^ + O_2_↑. (1)

**Figure 1:**
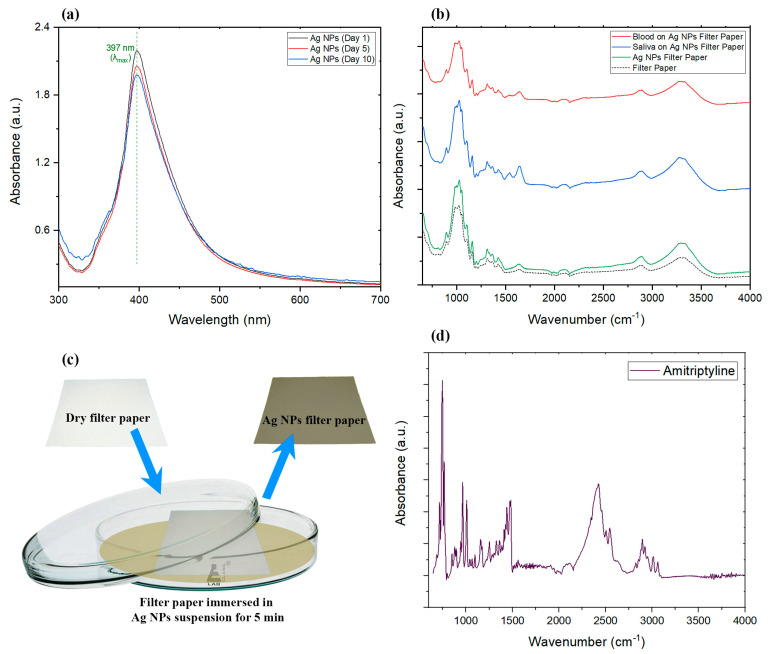
(**a**) UV/Vis spectra of fresh and aged silver nanoparticle suspension (λ_max_ = 397 nm). (**b**) Comparison of the FTIR results of filter paper with and without AgNPs, as well as dried blood and dried saliva on AgNP-decorated filter paper. (**c**) The decorating process of silver nanoparticles onto the filter paper sample. (**d**) FTIR spectrum of AMT and AgNPs in the suspension.

Drop-casting of silver nanoparticles on paper was previously illustrated [53]; however, in this study, we utilized a dipping method [55] to modify filter papers, which sped up the process of developing our three-dimensional (3D) enhanced surface and could be more suitable for large-scale commercial production compared to other techniques. Here, we provide a separate bath filled with developed AgNPs, and casting nanoparticles on the paper was performed by submerging filter paper in the solution for 5 min before removing and drying the now gray-colored paper (Figure 1c). The dried paper was then cut into 4 cm^2^ pieces for the experiments. The characteristics of the developed nanoparticles in the solution and on the paper were then studied using spectroscopy and microscopy techniques.

UV/Vis spectroscopy was used to confirm the production and stability of the silver nanoparticles in the suspension. To investigate the stability, 50 mL of synthesized AgNP suspension was separated right after synthesis ended and kept at room temperature while protected from the light. This batch of AgNPs was used to study the aging effects on nanoparticles using UV/Vis spectroscopy.

### 2.3. Biological Samples

Saliva samples were collected from a healthy adult volunteer and kept in the fridge until use. Various standard AMT solutions were added to saliva samples to develop artificially spiked samples at a desired concentrations in a range between 50 ppb and 1.75 ppm of AMT, considering the therapeutic range of AMT [3]. Then, 10 μL of each developed saliva sample was directly applied onto filter papers with and without AgNP decoration. A similar procedure was performed to obtain spiked blood samples in the range between 50 ppb and 1.75 ppm of AMT. Horse blood was used for this study, as both human and horse blood have relatively close Raman spectra and characteristics [61]; samples were stored in the fridge until use.

### 2.4. Results and Discussion

#### 2.4.1. FTIR and UV/Vis 

Paramelle et al. used the combination of high-resolution transmission electron microscopy (HR-TEM) and UV/Vis spectroscopy techniques to illustrate the exact correlation between the UV/Vis absorbance peak wavelength and the size of the developed nanoparticles [62]. They showed how the absorbance peak (λ_max_) for developed silver nanoparticles can appear in a range from 392 to 492 nm, which represents the increase in the average size of the developed nanoparticles in the suspension from 8 nm to 100 nm. The absorbance peak of the AgNPs we developed in this study appeared at 397 nm, which represents the formation of nanoparticles with a mean size of about 14 nm according to the Paramelle chart [62]. UV/Vis results were also utilized to monitor the change in the silver nanoparticles suspension over time. The developed silver suspension was kept in the dark, at approximately 20 °C for the period of 10 days, and it was tested at certain points to monitor the stability of the nanoparticles in the suspension developed for this experiment. As can be seen in Figure 1a, there was no significant change in the intensity or location of the peaks obtained on days 1, 5, and 10, which rules out the possibility of degradation, aggregation, or considerable precipitation of developed nanoparticles in the period of the experiment.

In order to demonstrate the sensitivity of Raman results, FTIR spectra of dried biological samples and filter papers were obtained for comparison. From the FTIR results in Figure 1b, it can be seen that the intensity of peaks was enhanced in the filter paper with silver nanoparticles compared to the paper without AgNPs, as expected [63,64]; however, the achieved infrared enhancement was not enough to be utilized for detecting trace amounts of AMT with FTIR. Most of the peaks were the same among all samples including CO vibration of cellulose at 1025.15 cm^−1^, symmetrical stretching vibration of COC in cellulose at 1162.72 cm^−1^, CH_2_ wagging in cellulose at 1312.79 cm^−1^, nonsymmetrical bending vibration of CH_3_ at 1433.43cm^−1^, weak OH bending peak at 1640.14 cm^−1^, CH_2_ stretching at 2891.42 cm^−1^, and OH stretching at 3308.52 cm^−1^ [65,66,67,68]. In addition to cellulose, peaks in the spectra represent other biomolecules such as proteins, lipids, nucleic acids, and carbohydrates in biological fluid. For instance, primary and secondary amide groups can be found in the region between 1500 cm^−1^ and 1700 cm^−1^, which are related to proteins and nucleic acids [69,70]. Lipids are also characterized by absorption peaks between 2970 cm^−1^ and 2850 cm^−1^, corresponding to asymmetric stretching vibrations of CH_3_ and CH_2_, and symmetric vibration of CH_2_. Phospholipids are characterized by FTIR absorption peaks at about 1060 and 1250 cm^−1^ [71,72], again observable in our spectra. Carbohydrates such as glucose are expected to be found in the regions of 990 to 1440 cm^−1^ and 2620 to 3060 cm^−1^ [73,74]. FTIR showed that pristine filter paper was mainly composed of carbon and oxygen. At the same time, it can be clearly seen that drying samples on the surface of the filter paper with silver modification reduced the intensity of the peaks, and this reduction was higher in the case of dried blood compared to dried saliva. The only exception to mention would be the presence of more intense OH bending peak in the dried saliva sample. Pristine AMT (Figure 1d) showed its fingerprint FTIR bands including the nitro compound peak at 1255.41 cm^−1^, CH stretching at 966.31 cm^−1^, CH bending at 756.66 cm^−1^, and C_6_H_6_ substitutions at 747.83 cm^−1^ and 764.75 cm^−1^ [75]. 

#### 2.4.2. SEM and EDAX

**Figure 2:**
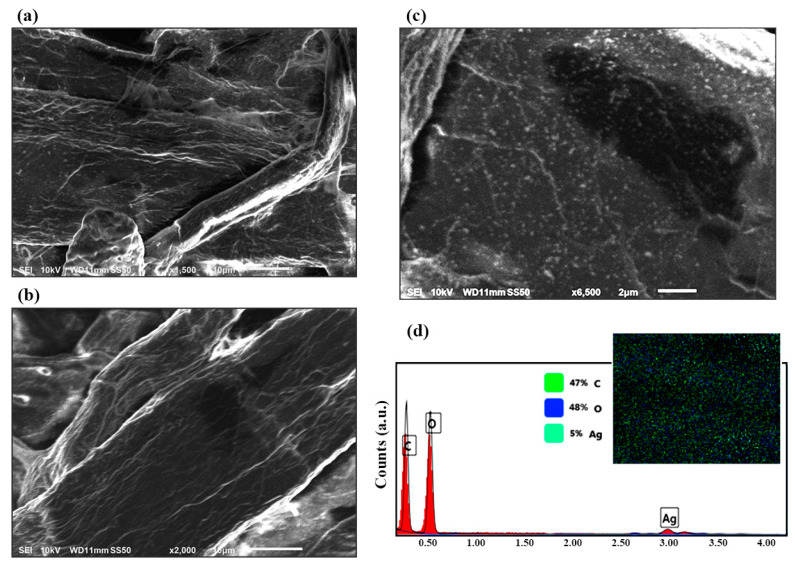
SEM micrographs of the paper after (**a**) and before (**b**) decorating the surface with silver nanoparticles. (**c**) Close examination of the AgNPs on the surface of the filter paper. (**d**) EDAX results of the decorated surface, confirming the presence of silver. Scale bars are 2 μm for (**c**) and 10 μm for (**a**,**b**).

SEM images were used compare the surface of the fabricated SERS substrate with clustered silver nanoparticles in the paper fiber pores (Figure 2a,c) with the surface of the substrate before the modification (Figure 2b). Figure 2c shows how silver decorated the fibers in the filter paper and how the dipping technique achieved a uniform surface coverage of nanoparticles. This silver nanoparticle clustering is responsible for the high SERS activity of the substrate [76]. The stable and homogenous adsorption of AgNPs on the fibers seen in the SEM results is a result of simple van der Waals interactions without the need for any specific chemical crosslinking procedures [53]. EDAX analysis (Figure 2d) of decorated filter paper also confirmed the presence of trace amounts of AgNPs all over the surface. From the elemental analysis graph and the element distribution pattern, it can be seen that the sample had approximately 47% mass of C, 47% mass of O, and 5% mass of Ag.

#### 2.4.3. SERS

**Figure 3:**
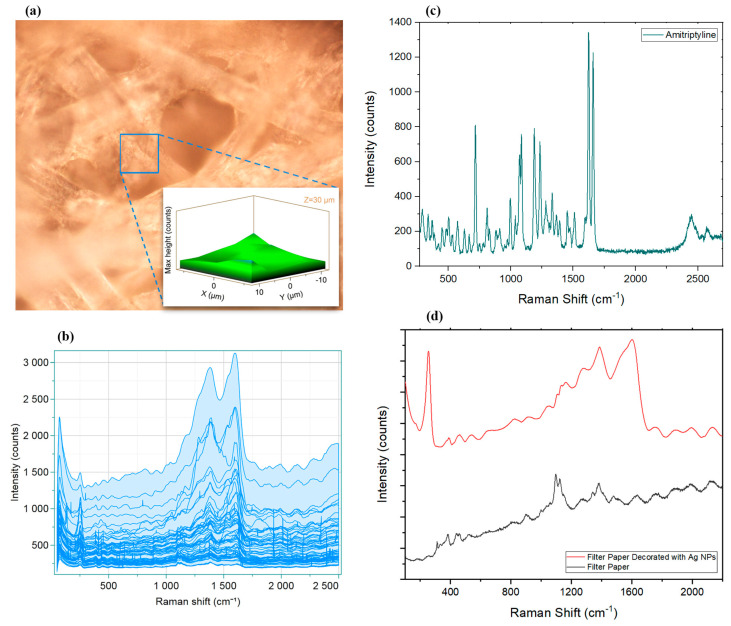
(**a**) SERS 3D mapping of a rectangular area of 20 μm (X-axis) × 20 μm (Y-axis) × 10 μm (Z-axis) of AgNP-decorated filter paper. (**b**) Data obtained from studying 125 data points in the same area, showing the entire hyperspectral dataset of spectra collected. Raman spectrum of pristine AMT crystals demonstrated in (**c**). (**d**) Raman spectra of filter paper before and after modification with AgNPs.

The Raman spectrum of pristine AMT crystals (Figure 3c) provided its specific fingerprint including the twisting six-membered ring (6MR) at 338.70 cm^−1^, in-plane bending seven-membered ring (7MR) at 504.91 cm^−1^, in-plane bending 6MR at 712.12 cm^−1^, CC stretching of 7MR at 994.12 cm^−1^, CH rocking of both 6MRs at 1068.38 cm^−1^ and 1083.34 cm^−1^, CC stretching of 7MR at 1186.92 cm^−1^ and 1231.80 cm^−1^, NC in-plane bending at 1451.20 cm^−1^, and C–C and C=C stretching at 1623.50 cm^−1^ and 1658.96 cm^−1^, respectively [77]. 

The paper-based SERS substrate can be cut into any desired shape, which improves the utilization of the SERS substrate. At the same time, it requires a much shorter waiting time for the sample droplet to dry, compared to other substrates, including conventional office paper, owing to the water conductivity of the filter paper, which makes the SERS detection faster and more convenient [78]. Figure 3d shows how adding AgNPs changed the Raman spectra of the filter paper. Comparing spectra before and after AgNP decoration (Figure 3d), it can be seen that AgNPs strengthened the intensities of most bands. However, the 1095.12 cm^−1^ band which originated from filter paper was masked by other SERS bands in the presence of AgNPs [79]. The strongest SERS peaks at 1604.04 cm^−1^, 1383.97 cm^−1^, and 254.37 cm^−1^ were attributed to the vibrations of silver nanoparticles (OCO, CC, and Ag–OCO) [80,81].

The detection of trace analytes requires suitable sensitivity and quantitative capability of the developed system, while another critical factor is the reproducibility of the signal in the presence of molecules of interest. In the case of SERS, to confirm the application of the substrate and prove the reproducibility of the signals, developing a uniform surface is key [82]. To evaluate these factors, Raman mapping of AgNP-decorated filter paper was obtained and analyzed (Figure 3a,b). A total of 125 points were analyzed in a well-focused section (50× objective; Figure 3a) in three dimensions (20 μm × 20 μm × 10 μm), which resulted in a 3D map that suggested a homogenous surface. Furthermore, calculating the relative standard deviation (RSD) of 40 scans of predominant peaks at 1604.04 cm^−1^, 1383.97 cm^−1^, and 254.37 cm^−1^ at different spots on the surface (fixed Z-axis) gave values of 10.10%, 8.34%, and 7.19%, respectively, revealing that the surface was homogenous and results were reproducible.

**Figure 4:**
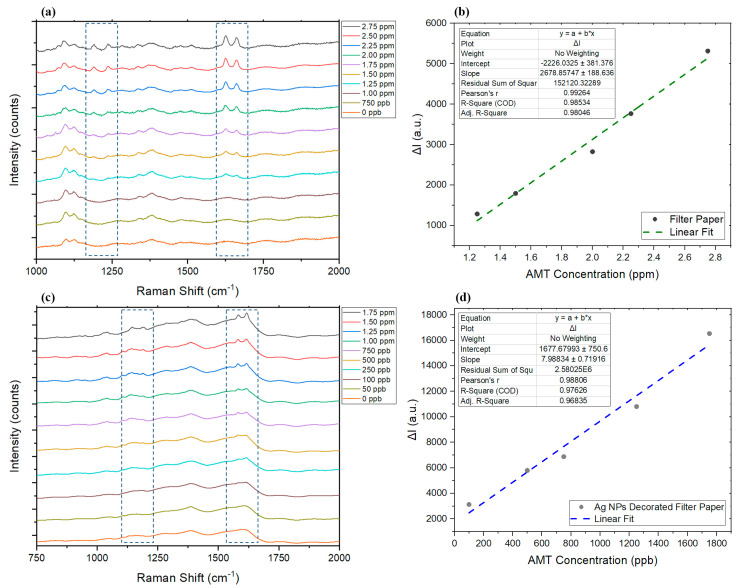
(**a**) Changes in the Raman spectra in the presence of different concentrations of AMT on the normal paper. (**b**) The linear range for each tested substrate. (**c**) The response of the AgNP-modified filter paper to changes in the concentration of AMT and (**d**) its linear range. ΔI represents the changes in intensity in the presence of AMT compared to the Raman intensity at the peak wavelength of blank sample.

To demonstrate the application of SERS for the detection of AMT and illustrate the advantages of developing an SERS substrate over normal substrate (pristine filter paper) and conventional Raman, 10 μL of AMT standards made in water (concentrations range between 50 ppb and 3 ppm) were added to both normal and silver-decorated filter paper and left to dry on the substrate at room temperature. A slight shift in the predominant Raman peaks occurred as expected [83] when studying the same standard sample on SERS substrate and conventional substrate (filter paper without AgNPs); however, the presence of two sharp peaks close to each other is still a good indicator of presence of AMT, especially since it can be seen that the intensity of the peaks increased upon adding more AMT to the sample (Figure 4a,c). In the Raman spectra of both substrates, we can see two groups of peaks that appeared upon increasing the concentration of AMT on the surface. While four peaks appeared and changed intensity depending on the concentration of the AMT, not all four might be suitable to monitor AMT concentration changes in a wide range from very low to high concentrations. The sharp twin peaks appearing between 1580 cm^−1^ and 1680 cm^−1^ in both substrates appeared at much lower concentrations and were still visible at high concentrations compared to the weaker twin peaks between 1140 cm^−1^ and 1240 cm^−1^. However, the latter group (weaker twin peaks) could still be used to confirm the presence of AMT when studying samples with relatively high concentrations. Limit of detection and linear range studies on the conventional filter paper were carried out as a function of the changes in intensity of the peak at about 1626 cm^−1^ in the presence of AMT, compared to a blank sample. The peak at about 1617 cm^−1^ was chosen to carry out the same study on AgNP-decorated filter paper. By comparing the Raman spectra (Figure 4a,c), it can be seen how presence of silver nanoparticles on the surface made it possible to identify the presence of much lower concentrations of AMT, which were not previously detectable on the normal substrate with SERS. The linear range and detection limits (LOD) for both samples were obtained as shown in Figure 4b,c. The linear range for normal substrate was found to be between 1.25 ppm and 2.75 ppm AMT (R^2^ = 0.98). The detection limit was determined from the lowest concentration of the AMT sample which produced a clearly detectable Raman scattering peak, which was found to be 1.20 ppm. On the other hand, upon increasing the AMT concentration on the filter paper (without AgNPs), we could still see an increase in the targeted peak intensities, until it reached the Raman saturation level, but the best linear response was found to be in a smaller range. However, the silver-decorated substrate was able to develop much more intense responses and resulted in generating detectable AMT-specific peaks at much lower concentrations to achieve a linear range between 100 ppb and 1.75 ppm AMT (R^2^ = 0.97). The lowest AMT concentration that was found to generate a detectable signal on the modified substrate was 95 ppb.

After investigating the possibility of sensing and measuring different concentrations of AMT on AgNP-decorated filter paper, it was necessary to confirm the applicability of the proposed sensing strategy, especially in the presence of various matrices. Two biological sample types, blood and saliva, both carrying natural large proteins and complex macromolecules, were selected to study the sensor response. In cases where conventional blood sampling is challenging or considered invasive, dried saliva spots and dried blood spots can provide a practical alternative for collecting and studying samples [84,85]. Dried spot tests can be collected remotely and studied later in the laboratory, in cases where a portable Raman spectrometer is not available. In the next sections, we evaluate the developed SERS method using a highly absorbent and modified substrate as a possible clinical toxicology technique to analyze AMT as an example of drugs that might be detectable using this technique. 

**Figure 5:**
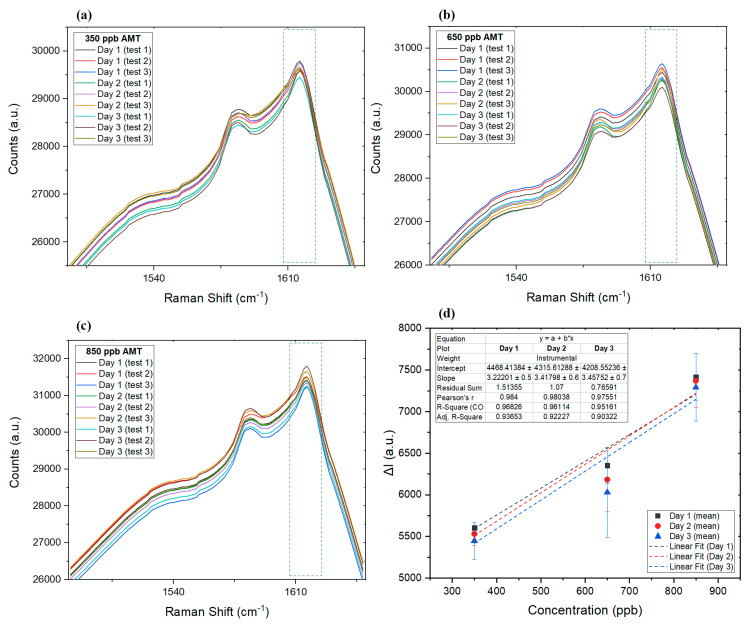
The Raman spectra of the of three tested standard samples of 350 ppb (**a**), 650 ppb (**b**), and 850 ppb (**c**). (**d**) Linear graph for the mean data collected on each day, along with error bars for each day.

While onsite Raman measurements using a portable Raman spectroscope is an option, in some cases, dried biological samples that are collected on the spot test substrates, might take some time to reach the laboratory for analysis. To study how this delay can affect the results of the utilized SERS technique, the intraday validation analysis was performed on the same samples in the period of 3 days. Our SERS protocol for AMT was applied in a validation study assessing the precision and accuracy of AMT in standards, as well as dried blood and dried saliva samples. AMT at three concentrations (350 ppb, 650 ppb, and 850 ppb) within the linear range of the detection method were run in triplicate each day, for a total of 3 days with accompanying blank samples, using our SERS protocol; the results are listed in Table 1. Figure 5a–c show how Raman spectra for each of the listed data in Table 1 changed over the course of the validation period. Furthermore, Figure 5d compares the changes in the regression and linear range of the sensor for the three selected concentration points, where the R^2^ factor starts at about 0.97 for day 1 and slowly reduces each day to 0.95 on day 3. The same decline pattern in R^2^ value is expected in real samples, which was later confirmed by comparing R^2^ on the first and third days of DBS and DSS tests.

Considering the low error reported in Table 1, it is expected that, even after 3 days, collected samples can still provide reliable measurements of AMT. It is expected that, when provided with a simple test kit including sterile filer paper, the silver suspension, and a sterile container to immerse and dry paper (or just an AgNP filter paper), even an untrained individual can easily collect samples (saliva or blood) at home and post them to a laboratory for SERS analysis. While silver nanoparticles make the detection of AMT at low concentrations with Raman possible, due to their antibacterial nature [86,87,88], they are expected to help with preserving biological samples longer.

Validation design consisted of the three AMT concentrations run in triplicate, plus a blank sample each day, with experiments repeated for three continuous days. The mean, RSD, and error for each concentration on daily experiments and across all experiments during the period of 3 days are provided in Table 1. Comparing the interday response of the SERS detection technique showed a minor analyte loss; however, comparing the intraday results suggests that the error slowly increased as a function of sample and substrate aging over time. This increase could have been caused by the slow degradation of silver nanoparticles during the experiments as they became exposed to light and temperature changes.

#### 2.4.4. Dried Blood

**Figure 6:**
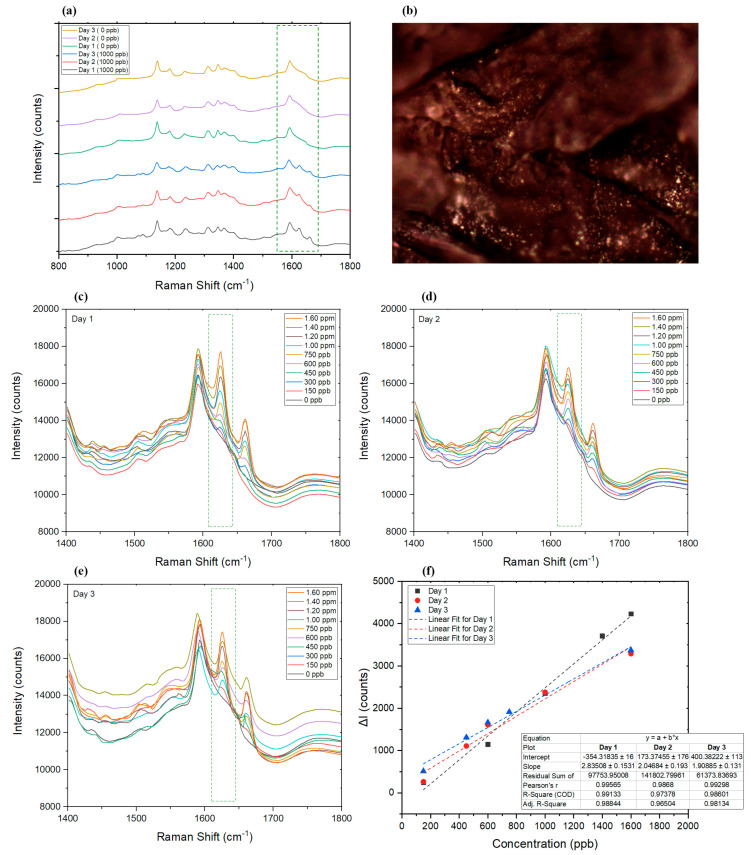
(**a**) SERS results of the dried blood sample with (1000 ppb) and without (0 ppb) AMT on days 1, 2, and 3. (**b**) Dried blood sample under the Raman microscope (50× objective). (**c**–**e**) Graphs representing the Raman spectra of samples on days 1, 2, and 3, respectively. (**f**) Comparison of the linear range of the utilized SERS technique on days 1, 2, and 3. ΔI represents the changes in intensity at the selected wavelength in the presence of AMT compared to the Raman intensity at the peak wavelength of blank sample (dried blood without AMT).

To investigate changes in the SERS results in the presence of different concentrations of AMT, 10 μL of spiked blood sample (within the linear range previously found for the AgNP filter paper) was applied on separate AgNP-decorated filter papers to study the limit of detection and linear range of the detection technique in dried blood. While the Raman spectra of all spiked blood samples were not exactly the same, due to the presence of different cells, proteins, and other biomolecules in the scan point [89] (Figure 6b), the selected peaks representing AMT still showed a linear change in correlation to the changes in AMT concentration. The range of the technique for dried blood samples was found to be between 150 ppb and 1.6 ppm AMT, as shown in Figure 6a,c,f.

##### Validation Parameters for Blood

There is a potential for aging of blood spots and the related stability of AMT to affect the detection of AMT by SERS. Blood chemistry and biomolecular changes can occur as a bloodstain ages [90], and, considering that dried blood spot samples may be collected remotely with a potential delay before analysis, it is important to study the potential for this to affect the validity of results. Hence, the effect of blood sample aging on the intensity of response and linear range of the technique was studied (Figure 6a,c–f). Figure 6a shows the spectra of dried blood in the presence and absence of AMT over a period of 3 days. The intensities of the fluorescence background profile of some of the samples increased on day 3 (Figure 6e) [91]; however, despite the changes in the background, the linear range on all 3 days remained quite consistent (Figure 6f), and R^2^ for the linear fit of the Raman spectra obtained on days 1, 2, and 3 was found to be 0.99, 0.97, and 0.98, respectively. This consistency in response to AMT, despite the slow changes in the matrix (i.e., blood), could also be due to the fact that AMT has a long lifetime and has scarcely photodegraded in the aqueous samples [92]; hence, AMT remains detectable in dried blood spots even after 3 days. For longer periods of time, due to the noticeable increase in the background spectra of blood, AMT spectra is masked by overlapping peaks. The validation results of analyzing dried blood control samples are summarized in Table 2.

#### 2.4.5. Dried Saliva

**Figure 7:**
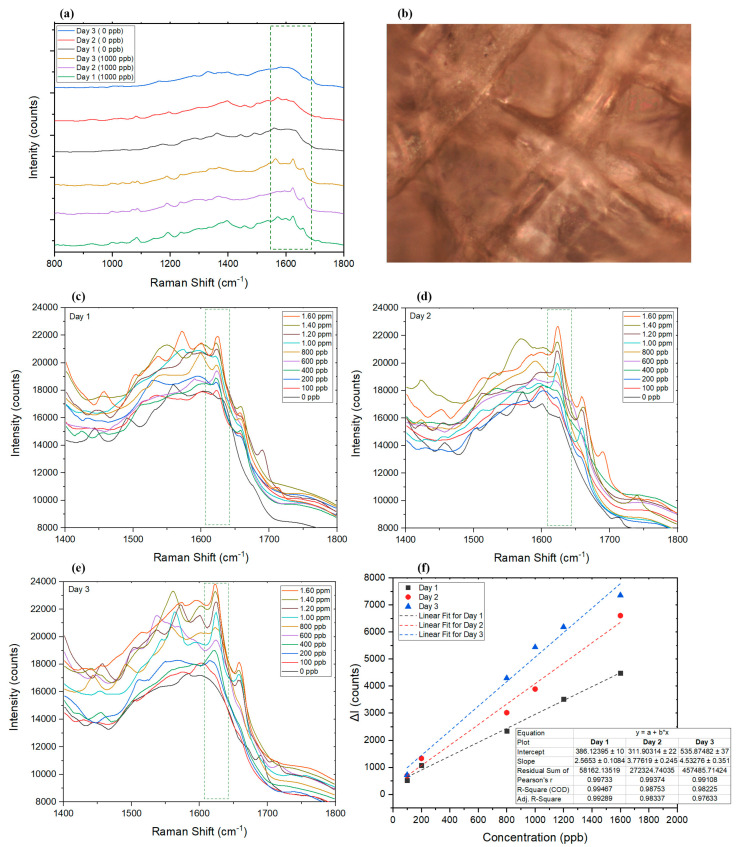
(**a**) SERS results of the dried saliva sample with (1000 ppb) and without (0 ppb) AMT on days 1, 2, and 3. (**b**) Dried saliva sample under Raman microscope (50× objective). (**c**–**e**) Graphs representing the Raman spectra of samples on days 1, 2, and 3, respectively. (**f**) Comparison of the linear range of the utilized SERS technique on days 1, 2, and 3. ΔI represents the changes in intensity at the selected wavelength in the presence of AMT compared to the Raman intensity at the peak wavelength of blank sample (dried saliva without AMT).

After collecting saliva from a healthy volunteer, 10 μL of the untreated saliva samples were used for SERS analysis to obtain the background spectrum of the dried saliva on the SERS substrate and compare the spectra of spiked samples. Results showed that dried saliva developed a less complicated spectrum (Figure 7a) than dried blood (Figure 6a), where it represented many similarities to the SERS substrate itself (Figure 4c). The Raman spectra for saliva did not interfere with AMT measurement. The selected AMT peak showed a linear change in correlation to the changes in AMT concentration in a range between 100 ppb and 1.6 ppm (Figure 7a,c,f). The wider linear range of the technique for dried saliva, compared to dried blood, was attributed to the simplicity of the saliva matrix compared to blood. Similar to blood, saliva is also considered a relatively complex biofluid holding proteins, metabolites, carbohydrates, nucleic acids, and hormones [93] released from oral cavity cells and salivary glands [94]. Saliva sample collection is considered to be completely noninvasive [95,96,97], and it can be a useful alternative to blood or urine samples which are more invasive in nature (assuming the detection window requirement is as required) [47].

##### Validation Parameters for Saliva

The effect of aging dried saliva samples has also been investigated up to 3 days after the samples were collected on the AgNP substrate (Figure 7a,c). Figure 7a compares the Raman spectra of dried saliva samples in the presence and absence of AMT on three different days. It can be seen that the intensity of AMT peak strengthened each day, which was assumed to be related to the degradation of molecules in saliva that allowed AMT a better contact with AgNPs to enhance the signal response, while AMT molecules remained almost intact [92] (Figure 7f). The R^2^ factor calculated for the linear fit of the Raman spectra was calculated to be 0.99, 0.98, and 0.98, on days 1, 2, and 3, respectively. For periods longer than 3 days, fewer samples were found to follow a linear pattern. The results of analyzing dried saliva control samples are summarized in Table 3.

### 2.5. Performance Comparisons of the Detection of Amitriptyline with Other Methods

Table 4 provides a comparison of the developed method in this paper with previously reported analytical methods for the determination of AMT. As can be seen in Table 4, while using DBS to measure AMT by chromatography techniques has previously been investigated [3,4,98,99], utilizing DSS to analyze AMT concentrations has not been reported to date. Electrochemical detection techniques can offer significantly faster response [2,100,101,102] compared to any other reported chromatography techniques. Despite the rapid nature of these techniques, none of the electrochemical sensors have previously been used to analyze dried biological samples. Considering the nature of electrochemical cells that commonly require an electrolyte, dried samples such as DBS and DSS need an extra pretreatment step before they can be analyzed with such techniques, which affects the length and costs of the experiments. SERS, on the other hand, can not only be operated without any sample pretreatments, but can also provide results as quickly as electrochemical techniques and overcome limitations faced in both chromatography and electrochemistry. Table 4 shows how our suggested method does not require a pretreatment step and offers rapid measurement of AMT in dried biological samples, while providing a reasonable detection limit and offering a wide linear range compared to previously developed detection methods. 

## 3. Conclusions

In this work, we developed a highly sensitive 3D filter paper substrate for SERS trace AMT detection in dried biological samples. The simple SERS-active filter paper was developed to aid the measurement of amitriptyline in complex samples on surfaces. Developed silver nanoparticles showed good stability in suspension form and were long-lasting, making it possible to provide a simple test kit (consisting of sterile filer paper, a silver suspension, and a sterile container to immerse and dry paper), whereby even an untrained individual can easily collect samples at home and post them to a laboratory for SERS analysis. Replacing the filter paper base with a more homogenous surface, before addition of silver nanoparticles, is likely to further improve the reproducibility of the results; however, filter paper used in this experiment offers much better liquid absorbance capacity, shorter drying time, and affordability, compared to other replacements. The quantitative and sensitive nature of the developed substrates was demonstrated by successfully measuring low concentrations of amitriptyline in dried blood and saliva samples. While dried samples can be collected on site and transferred to the laboratory later, it is also possible to use portable Raman analyzers to provide rapid onsite results, which can be beneficial for pharmaceutical, toxicological, crime scene, and forensic studies. Modifying conventional dried spot tests with AgNPs can enhance the speed and sensitivity of the testing process when combined with SERS. 

## Figures and Tables

**Table 1:** Precision and accuracy of controls in standards.

Type of Precision		Spiked Amount of AMT (ppb)	350	650	850
		Analytical Variables			
Intraday	Day 1	Number of experiments	3	3	3
		Mean	341.366	622.985	814.943
		Relative standard deviation	3.048%	2.017%	4.121%
		Error	2.467%	4.156%	4.124%
	Day 2	Number of experiments	3	3	3
		Mean	332.163	601.582	809.192
		Relative standard deviation	3.438%	2.473%	2.539%
		Error	5.096%	7.449%	4.801%
	Day 3	Number of experiments	3	3	3
		Mean	321.797	582.324	799.176
		Relative standard deviation	4.198%	2.291%	2.201%
		Error	8.058%	10.412%	5.979%
Interday		Number of experiments	9	9	9
		Mean	331.775	602.297	807.770
		Relative standard deviation	4.012%	3.521%	2.803%
		Error	5.207%	7.339%	4.968%

**Table 2:** Precision and accuracy of controls in dried blood samples.

Spiked Amount of AMT	350 ppb		650 ppb		850 ppb	
	Intraday	Interday	Intraday	Interday	Intraday	Interday
Number of experiments	3	9	3	9	3	9
Mean	338.11	332.02	612.15	609.20	818.770	801.04
Relative standard deviation	2.030%	3.964%	3.015%	3.149%	3.511%	4.472%
Error	3.397%	5.137%	5.823%	6.277%	3.674%	5.760%

**Table 3:** Precision and accuracy of controls in dried saliva samples.

Spiked Amount of AMT	350 ppb		650 ppb		850 ppb	
	Intraday	Interday	Intraday	Interday	Intraday	Interday
Number of experiments	3	9	3	9	3	9
Mean	329.27	334.17	609.16	598.90	802.10	813.52
Relative standard deviation	4.012%	8.330%	4.251%	13.727%	3.618%	15.204%
Error	5.923%	4.523%	6.283%	7.862%	5.635%	4.292%

**Table 4:** Performance and comparison of AMT detection techniques.

Technique	Sensing Material	LOD	Linear Range	Run Time	Pre-Treatment	Real Sample (s)	Ref.
LSV ^1^	Plasticized PVC membrane-coated GCE	3 nM	3 nM–3 μM	<1 min	None	Bovine serum albumin, whole blood	[100]
CV ^2^	GO/Fe_3_O_4_@SiO_2_/GSPE	0.5 μM	2 μM–1 mM	<1 min	None	Urine, medical tablets	[101]
CV	Phosphorus-doped MWCNTs/GCE	0.15 μg/mL	0.5 μg/mL–40 μg/mL	<1 min	None	Urine	[102]
CV	rGO/7′-methoxy-[1,1′-binaphthalen]-7-ol	1 ng/mL	1 ng/mL–80 μg/mL	<1 min	None	NA	[2]
LC–MS/MS	---	10.4 μg/mL	20 μg/mL–500 μg/mL	4.8 min	None	DBS	[4]
LC–HRMS ^3^	---	10 ng/mL	5 ng/mL–200 ng/mL	12.5 min	>4 min	DBS	[98]
HPLC–MS	---	3.91 μg/mL	3.91 μg/mL–500 μg/mL	23 min	50 min	DBS	[99]
CE–MS ^4^	---	1.76 ng/mL	1.76 ng/mL–300 ng/mL	NA	3 h	DBS	[3]
SERS	AgNP-decorated cellulose filter paper	95 ng/mL	100 ng/mL–1.75 μg/mL	<1 min	None	DBS, DSS	This work

^1^ Linear sweep voltammetry; ^2^ cyclic voltammetry; ^3^ liquid chromatography/high-resolution mass spectrometry; ^4^ capillary electrophoresis/mass spectrometry.

## References

[1] Bryson H.M., Wilde M.I.J.D. (1996). Amitriptyline. Drugs Aging.

[2] Boroujerdi R., Abdelkader A., Paul R. (2022). Highly sensitive and selective detection of the antidepressant amitriptyline using functionalised graphene-based sensor. ChemNanoMat.

[3] Świądro M., Stelmaszczyk P., Wietecha-Posłuszny R., Dudek D. (2020). Development of a new method for drug detection based on a combination of the dried blood spot method and capillary electrophoresis. J. Chromatogr. B.

[4] Berm E.J., Paardekooper J., Brummel-Mulder E., Hak E., Wilffert B., Maring J.G.T. (2015). A simple dried blood spot method for therapeutic drug monitoring of the tricyclic antidepressants amitriptyline, nortriptyline, imipramine, clomipramine, and their active metabolites using LC-MS/MS. Talanta.

[5] Van Brunt N. (1983). The clinical utility of tricyclic antidepressant blood levels: A review of the literature. Ther. Drug Monit..

[6] Qiu X., Chen C., Shi Y., Chen K., Li M., Xu H., Wu X., Takai Y., Shimasaki Y., Oshima Y. (2022). Persistent impact of amitriptyline on the behavior, brain neurotransmitter, and transcriptional profile of zebrafish (*Danio rerio*). Aquat. Toxicol..

[7] Abdollahi M., Mostafalou S., Wexler P. (2014). Tricyclic Antidepressants. Encyclopedia of Toxicology.

[8] Yang K.L., Dantzker D.R. (1991). Reversible Brain Death: A Manifestation of Amitriptyline Overdose. Chest.

[9] Coull D.C., Dingwall-Fordyce I., Crooks J., Scott A.M., Weir R.D. (1970). Amitriptyline and Cardiac Disease: Risk of Sudden Death Identified by Monitoring System. Lancet.

[10] Moir D.C., Cornwell W.B., Dingwall-Fordyce I., Crooks J., O’Malley K., Turnbull M.J., Weir R.D. (1972). Cardiotoxicity of Amitriptyline. Lancet.

[11] Prahlow J.A., Landrum J.E. (2005). Amitriptyline abuse and misuse. Am. J. Forensic Med. Pathol..

[12] Boroujerdi R., Paul R. (2022). Graphene-Based Electrochemical Sensors for Psychoactive Drugs. Nanomaterials.

[13] DiMaio V., DiMaio D. (2001). Interpretive toxicology: Drug abuse and drug deaths. Forensic Pathol..

[14] Garrido-Suárez B.B., Garrido G., Bellma Menéndez A., Merino N., Valdés O., Delgado-Hernández R., Granados-Soto V. (2021). Synergistic interaction between amitriptyline and paracetamol in persistent and neuropathic pain models: An isobolografic analysis. Neurochem. Int..

[15] Blahova J., Doubkova V., Plhalova L., Lakdawala P., Medkova D., Vecerek V., Svobodova Z., Faggio C. (2021). Embryotoxicity of selective serotonin reuptake inhibitors—Comparative sensitivity of zebrafish (*Danio rerio*) and african clawed frog (xenopus laevis) embryos. Appl. Sci..

[16] Richmond E.K., Rosi E.J., Walters D.M., Fick J., Hamilton S.K., Brodin T., Sundelin A., Grace M.R. (2018). A diverse suite of pharmaceuticals contaminates stream and riparian food webs. Nat. Commun..

[17] Bezerra R.D., Morais A.I., Osajima J.A., Nunes L.C., Silva Filho E.C. (2016). Development of new phosphated cellulose for application as an efficient biomaterial for the incorporation/release of amitriptyline. Int. J. Biol. Macromol..

[18] Chen Y., Xiao M., Wang Z., Jiang W., Guo Y., Liu Z. (2016). Oxidation of amitriptyline and nortriptyline by ferrate (VI): Efficiency and reaction pathways. Desalin. Water Treat..

[19] Melin V., Salgado P., Thiam A., Henríquez A., Mansilla H.D., Yáñez J., Salazar C. (2021). Study of degradation of amitriptyline antidepressant by different electrochemical advanced oxidation processes. Chemosphere.

[20] Guthrie R., Susi A. (1963). A simple phenylalanine method for detecting phenylketonuria in large populations of newborn infants. Pediatrics.

[21] Bezerra C.S., Portilho M.M., Barbosa J.R., de Azevedo C.P., Mendonça A.C.d.F., da Cruz J.N.M., Frota C.C., do Lago B.V., Villar L.M. (2022). Dried blood spot sampling for hepatitis B virus quantification, sequencing and mutation detection. Sci. Rep..

[22] Warszawski J., Beaumont A.-L., Seng R., de Lamballerie X., Rahib D., Lydié N., Slama R., Durrleman S., Raynaud P., Sillard P. (2022). Prevalence of SARS-CoV-2 antibodies and living conditions: The French national random population-based EPICOV cohort. BMC Infect. Dis..

[23] Thevis M., Kuuranne T., Geyer H. (2022). Annual banned-substance review: Analytical approaches in human sports drug testing 2020/2021. Drug Test. Anal..

[24] Li W., Tse F.L. (2010). Dried blood spot sampling in combination with LC-MS/MS for quantitative analysis of small molecules. Biomed. Chromatogr..

[25] Edelbroek P.M., van der Heijden J., Stolk L.M. (2009). Dried blood spot methods in therapeutic drug monitoring: Methods, assays, and pitfalls. Ther. Drug Monit..

[26] La Marca G., Malvagia S., Filippi L., Luceri F., Moneti G., Guerrini R. (2009). A new rapid micromethod for the assay of phenobarbital from dried blood spots by LC-tandem mass spectrometry. Epilepsia.

[27] Parker S., Cubitt W.D. (1999). The use of the dried blood spot sample in epidemiological studies. J. Clin. Pathol..

[28] Iacuzzi V., Posocco B., Zanchetta M., Gagno S., Poetto A.S., Guardascione M., Toffoli G. (2021). Dried Blood Spot Technique Applied in Therapeutic Drug Monitoring of Anticancer Drugs: A Review on Conversion Methods to Correlate Plasma and Dried Blood Spot Concentrations. Pharm. Res..

[29] Hansen S.H., Pedersen-Bjergaard S., Rasmussen K. (2011). Introduction to Pharmaceutical Chemical Analysis.

[30] Breyer-Pfaff U. (2004). The metabolic fate of amitriptyline, nortriptyline and amitriptylinoxide in man. Drug Metab. Rev..

[31] Langman L.J. (2007). The use of oral fluid for therapeutic drug management: Clinical and forensic toxicology. Ann. N. Y. Acad. Sci..

[32] Brodie B.B. (1967). Physicochemical and biochemical aspects of pharmacology. Jama.

[33] Elmongy H., Abdel-Rehim M. (2016). Saliva as an alternative specimen to plasma for drug bioanalysis: A review. TrAC Trends Anal. Chem..

[34] Almeida E., Soares S., Gonçalves J., Rosado T., Fernández N., Rodilla J.M., Passarinha L.A., Barroso M., Gallardo E. (2022). Stability of Cocaine, Opiates, and Metabolites in Dried Saliva Spots. Molecules.

[35] Soares S., Rosado T., Barroso M., Gallardo E. (2021). New method for the monitoring of antidepressants in oral fluid using dried spot sampling. Pharmaceuticals.

[36] Han Y., Li X.-L., Zhang M., Wang J., Zeng S., Min J.Z. (2021). Potential use of a dried saliva spot (DSS) in therapeutic drug monitoring and disease diagnosis. J. Pharm. Anal..

[37] Arntson A., Ofsa B., Lancaster D., Simon J.R., McMullin M., Logan B. (2013). Validation of a novel immunoassay for the detection of synthetic cannabinoids and metabolites in urine specimens. J. Anal. Toxicol..

[38] Nieddu M., Trignano C., Burrai L., Pirisi M.A., Boatto G. (2013). Cross-reactivities of 41 new amphetamine designer drugs to EMIT^®^ immunoassays. Forensic Toxicol..

[39] Mostowtt T., McCord B. (2017). Surface enhanced Raman spectroscopy (SERS) as a method for the toxicological analysis of synthetic cannabinoids. Talanta.

[40] Krieg A.K., Gauglitz G. (2015). Ultrasensitive label-free immunoassay for optical determination of amitriptyline and related tricyclic antidepressants in human serum. Anal. Chem..

[41] Barnes A.J., Young S., Spinelli E., Martin T.M., Klette K.L., Huestis M.A. (2014). Evaluation of a homogenous enzyme immunoassay for the detection of synthetic cannabinoids in urine. Forensic Sci. Int..

[42] Grüner N., Stambouli O., Ross R.S. (2015). Dried blood spots-preparing and processing for use in immunoassays and in molecular techniques. JoVE (J. Vis. Exp.).

[43] Dronova M., Smolianitski E., Lev O. (2016). Electrooxidation of new synthetic cannabinoids: Voltammetric determination of drugs in seized street samples and artificial saliva. Anal. Chem..

[44] Wolff K., Agombar R., Clatworthy A., Cowan D., Forrest R., Osselton D., Scott-Ham M., Johnston A. (2017). Expert Panel Review of Alternative Biological Matrices for Use as an Evidential Sample for Drug Driving.

[45] Williams D., Sebastine I. (2005). Tissue engineering and regenerative medicine: Manufacturing challenges. IEE Proc. Nanobiotechnol..

[46] Farquharson S., Shende C., Sengupta A., Huang H., Inscore F. (2011). Rapid detection and identification of overdose drugs in saliva by surface-enhanced Raman scattering using fused gold colloids. Pharmaceutics.

[47] Dana K., Shende C., Huang H., Farquharson S. (2015). Rapid analysis of cocaine in saliva by surface-enhanced Raman spectroscopy. J. Anal. Bioanal. Tech..

[48] Feng S., Huang S., Lin D., Chen G., Xu Y., Li Y., Huang Z., Pan J., Chen R., Zeng H. (2015). Surface-enhanced Raman spectroscopy of saliva proteins for the noninvasive differentiation of benign and malignant breast tumors. Int. J. Nanomed..

[49] Sharma B., Frontiera R.R., Henry A.-I., Ringe E., Van Duyne R.P. (2012). SERS: Materials, applications, and the future. Mater. Today.

[50] Rodriguez R.D., Sheremet E., Nesterov M., Moras S., Rahaman M., Weiss T., Hietschold M., Zahn D.R. (2018). Aluminum and copper nanostructures for surface-enhanced Raman spectroscopy: A one-to-one comparison to silver and gold. Sens. Actuators B Chem..

[51] He S., Chua J., Tan E.K.M., Kah J.C.Y. (2017). Optimizing the SERS enhancement of a facile gold nanostar immobilized paper-based SERS substrate. RSC Adv..

[52] Zhang W., Liu J., Niu W., Yan H., Lu X., Liu B.J. (2018). Tip-selective growth of silver on gold nanostars for surface-enhanced raman scattering. ACS Appl. Mater. Interfaces.

[53] Oliveira M.J., Quaresma P., Peixoto de Almeida M., Araújo A., Pereira E., Fortunato E., Martins R., Franco R., Águas H. (2017). Office paper decorated with silver nanostars-an alternative cost effective platform for trace analyte detection by SERS. Sci. Rep..

[54] Fierro-Mercado P.M., Hernández-Rivera S.P. (2012). Highly sensitive filter paper substrate for SERS trace explosives detection. Int. J. Spectrosc..

[55] Ngo Y.H., Li D., Simon G.P., Garnier G. (2012). Gold nanoparticle–paper as a three-dimensional surface enhanced Raman scattering substrate. Langmuir.

[56] Nguyen B.H., Nguyen V.H., Tran H.N. (2016). Rich variety of substrates for surface enhanced Raman spectroscopy. Nanotechnol. Adv. Nat. Sci. Nanosci. Nanotechnol..

[57] Mosier-Boss P.A. (2017). Review of SERS substrates for chemical sensing. Nanomaterials.

[58] Živanović V., Madzharova F., Heiner Z., Arenz C., Kneipp J. (2017). Specific interaction of tricyclic antidepressants with gold and silver nanostructures as revealed by combined one-and two-photon vibrational spectroscopy. J. Phys. Chem. C.

[59] Sun F., Hung H.-C., Sinclair A., Zhang P., Bai T., Galvan D.D., Jain P., Li B., Jiang S., Yu Q. (2016). Hierarchical zwitterionic modification of a SERS substrate enables real-time drug monitoring in blood plasma. Nat. Commun..

[60] Lee P., Meisel D. (1982). Adsorption and surface-enhanced Raman of dyes on silver and gold sols. J. Phys. Chem..

[61] McLaughlin G., Doty K.C., Lednev I.K. (2014). Discrimination of human and animal blood traces via Raman spectroscopy. Forensic Sci. Int..

[62] Paramelle D., Sadovoy A., Gorelik S., Free P., Hobley J., Fernig D.G. (2014). A rapid method to estimate the concentration of citrate capped silver nanoparticles from UV-visible light spectra. Analyst.

[63] Bibikova O., Haas J., López-Lorente Á.I., Popov A., Kinnunen M., Ryabchikov Y., Kabashin A., Meglinski I., Mizaikoff B. (2017). Surface enhanced infrared absorption spectroscopy based on gold nanostars and spherical nanoparticles. Anal. Chim. Acta.

[64] Kurrey R., Deb M.K., Shrivas K. (2019). Surface enhanced infra-red spectroscopy with modified silver nanoparticles (AgNPs) for detection of quaternary ammonium cationic surfactants. New J. Chem..

[65] Zhang K., Wang M., Wu M., Wu Q., Liu J., Yang J., Zhang J. (2020). Fabrication of robust superhydrophobic filter paper for oil/water separation based on the combined octadecanoyl chain bonding and polymer grafting via surface-initiated ATRP. Cellulose.

[66] Ou R., Xie Y., Shen X., Yuan F., Wang H., Wang Q. (2012). Solid biopolymer electrolytes based on all-cellulose composites prepared by partially dissolving cellulosic fibers in the ionic liquid 1-butyl-3-methylimidazolium chloride. J. Mater. Sci..

[67] Yu P., Yu H., Sun Q., Ma B. (2019). Filter paper supported nZVI for continuous treatment of simulated dyeing wastewater. Sci. Rep..

[68] Wu L.M., Tong D.S., Zhao L.Z., Yu W.H., Zhou C.H., Wang H. (2014). Fourier transform infrared spectroscopy analysis for hydrothermal transformation of microcrystalline cellulose on montmorillonite. Appl. Clay Sci..

[69] Barth A. (2007). Infrared spectroscopy of proteins. Biochim. Biophys. Acta Bioenerg..

[70] Surewicz W.K., Mantsch H.H., Chapman D. (1993). Determination of protein secondary structure by Fourier transform infrared spectroscopy: A critical assessment. Biochemistry.

[71] Dovbeshko G.I., Gridina N.Y., Kruglova E.B., Pashchuk O.P. (2000). FTIR spectroscopy studies of nucleic acid damage. Talanta.

[72] Góralski P. (1996). Interactions between cholesterol and triacylglycerols in carbon tetrachloride: Calorimetric and spectroscopic studies. Thermochim. Acta.

[73] Jessen T.E., Höskuldsson A.T., Bjerrum P.J., Verder H., Sørensen L., Bratholm P.S., Christensen B., Jensen L.S., Jensen M.A.B. (2014). Simultaneous determination of glucose, triglycerides, urea, cholesterol, albumin and total protein in human plasma by Fourier transform infrared spectroscopy: Direct clinical biochemistry without reagents. Clin. Biochem..

[74] Rohman A., Windarsih A., Lukitaningsih E., Rafi M., Betania K., Fadzillah N.A. (2019). The use of FTIR and Raman spectroscopy in combination with chemometrics for analysis of biomolecules in biomedical fluids: A review. Biomed. Spectrosc. Imaging.

[75] Kalyani G., Adjuad B., Baghel D.B.D., Sahu C., Singh R. (2016). Formulation and in-vitro evaluation of sustained release matrix tablet of amitriptyline. World J. Pharm. Pharm. Sci..

[76] Hoppmann E.P., Yu W.W., White I.M. (2013). Highly sensitive and flexible inkjet printed SERS sensors on paper. Methods.

[77] Jaworska A., Malek K. (2014). A comparison between adsorption mechanism of tricyclic antidepressants on silver nanoparticles and binding modes on receptors. Surface-enhanced Raman spectroscopy studies. J. Colloid Interface Sci..

[78] Lin S., Lin X., Lou X.-T., Yang F., Lin D.-Y., Lu Z.W. (2014). Rapid fabrication of self-assembled interfacial film decorated filter paper as an excellent surface-enhanced Raman scattering substrate. Anal. Methods.

[79] Wu D., Fang Y. (2003). The adsorption behavior of p-hydroxybenzoic acid on a silver-coated filter paper by surface enhanced Raman scattering. J. Colloid Interface Sci..

[80] Joshi N., Jain N., Pathak A., Singh J., Prasad R., Upadhyaya C.P. (2018). Biosynthesis of silver nanoparticles using Carissa carandas berries and its potential antibacterial activities. J. Sol-Gel Sci. Technol..

[81] Cai Y., Piao X., Gao W., Zhang Z., Nie E., Sun Z. (2017). Large-scale and facile synthesis of silver nanoparticles via a microwave method for a conductive pen. RSC Adv..

[82] Tan C., Zhang Z., Qu Y., He L. (2017). Ag_2_O/TiO_2_ nanocomposite heterostructure as a dual functional semiconducting substrate for SERS/SEIRAS application. Langmuir.

[83] Chong N.S., Smith K.A., Setti S., Ooi B.G. (2013). Application of gold and silver colloidal nanoparticles for the surface-enhanced Raman spectrometric analysis of melamine and 4-aminobiphenyl. Int. J. Environ. Technol. Manag..

[84] Binks M.J., Bleakley A.S., Rathnayake G., Pizzutto S., Chang A.B., McWhinney B., Ungerer J. (2021). Can dried blood spots be used to accurately measure vitamin D metabolites?. Clin. Chim. Acta.

[85] Han Y., Shi Q., Xu C.-Y., Di L., Zhao L.-L., Jin W., Min J.Z. (2021). A convenient sampling and noninvasive dried spot method of uric acid in human saliva: Comparison of serum uric acid value and salivary uric acid in healthy volunteers and hyperuricemia patients. J. Chromatogr. B.

[86] Franci G., Falanga A., Galdiero S., Palomba L., Rai M., Morelli G., Galdiero M. (2015). Silver nanoparticles as potential antibacterial agents. Molecules.

[87] Tang S., Zheng J. (2018). Antibacterial activity of silver nanoparticles: Structural effects. Adv. Healthc. Mater..

[88] Baker C., Pradhan A., Pakstis L., Pochan D.J., Shah S.I. (2005). Synthesis and antibacterial properties of silver nanoparticles. J. Nanosci. Nanotechnol..

[89] Atkins C.G., Buckley K., Blades M.W., Turner R.F. (2017). Raman spectroscopy of blood and blood components. Appl. Spectrosc..

[90] Doty K.C., Muro C.K., Lednev I.K. (2017). Predicting the time of the crime: Bloodstain aging estimation for up to two years. Forensic Chem..

[91] Takamura A., Watanabe D., Shimada R., Ozawa T. (2019). Comprehensive modeling of bloodstain aging by multivariate Raman spectral resolution with kinetics. Commun. Chem..

[92] Chen Y., Liang J., Liu L., Lu X., Deng J., Pozdnyakov I.P., Zuo Y. (2017). Photosensitized degradation of amitriptyline and its active metabolite nortriptyline in aqueous fulvic acid solution. J. Environ. Qual..

[93] Carlomagno C., Banfi P., Gualerzi A., Picciolini S., Volpato E., Meloni M., Lax A., Colombo E., Ticozzi N., Verde F. (2020). Human salivary Raman fingerprint as biomarker for the diagnosis of Amyotrophic Lateral Sclerosis. Sci. Rep..

[94] Chiappin S., Antonelli G., Gatti R., Elio F. (2007). Saliva specimen: A new laboratory tool for diagnostic and basic investigation. Clin. Chim. Acta.

[95] Qiu S., Xu Y., Huang L., Zheng W., Huang C., Huang S., Lin J., Lin D., Feng S., Chen R. (2016). Non-invasive detection of nasopharyngeal carcinoma using saliva surface-enhanced Raman spectroscopy. Oncol. Lett..

[96] Ma L., Zhang Z., Li X. (2020). Non-invasive disease diagnosis using surface-enhanced Raman spectroscopy of urine and saliva. Appl. Spectrosc. Rev..

[97] Zamora-Mendoza B., Espinosa-Tanguma R., Ramírez-Elías M., Cabrera-Alonso R., Montero-Moran G., Portales-Pérez D., Rosales-Romo J., Gonzalez J., Gonzalez C. (2019). Surface-enhanced raman spectroscopy: A non invasive alternative procedure for early detection in childhood asthma biomarkers in saliva. Photodiagnosis Photodyn. Ther..

[98] Joye T., Sidibé J., Déglon J., Karmime A., Sporkert F., Widmer C., Favrat B., Lescuyer P., Augsburger M., Thomas A. (2019). Liquid chromatography-high resolution mass spectrometry for broad-spectrum drug screening of dried blood spot as microsampling procedure. Anal. Chim. Acta.

[99] Stern M., Giebels M., Fey T., Lübking M., Alferink J., Hempel G. (2020). Validation and clinical application of a volumetric absorptive microsampling method for 14 psychiatric drugs. Bioanalysis.

[100] Guzinski M., Lindner E., Pendley B., Chaum E. (2022). Electrochemical sensor for tricyclic antidepressants with low nanomolar detection limit: Quantitative Determination of Amitriptyline and Nortriptyline in blood. Talanta.

[101] Zoheira Bagheri N., Fariba Garkani N. (2022). Electrochemical Sensor Based on a Modified Graphite Screen Printed Electrode for Amitriptyline Determination. Surf. Eng. Appl. Electrochem..

[102] Sanguarnsak C., Promsuwan K., Saichanapan J., Soleh A., Saisahas K., Phua C.H., Limbut W. (2022). Voltammetric Amitriptyline Determination Using a Metal-Free Electrode Based on Phosphorus-Doped Multi-Walled Carbon Nanotubes. J. Electrochem. Soc..

